# Impact of Sarcopenia on Functional Outcomes Among Patients With Mild Acute Ischemic Stroke and Transient Ischemic Attack: A Retrospective Study

**DOI:** 10.3389/fneur.2022.841945

**Published:** 2022-03-15

**Authors:** Hyungwoo Lee, Il Hyung Lee, JoonNyung Heo, Minyoul Baik, Hyungjong Park, Hye Sun Lee, Hyo Suk Nam, Young Dae Kim

**Affiliations:** ^1^Department of Neurology, Yonsei University College of Medicine, Seoul, South Korea; ^2^Department of Neurology, Keimyung University School of Medicine, Daegu, South Korea; ^3^Biostatistics Collaboration Unit, Department of Research Affairs, Yonsei University College of Medicine, Seoul, South Korea; ^4^Integrative Research Center for Cerebrovascular and Cardiovascular Diseases, Yonsei University College of Medicine, Seoul, South Korea

**Keywords:** sarcopenia, stroke, prognosis, muscle, aged

## Abstract

**Introduction:**

Sarcopenia, a age-related disease characterized by loss of muscle mass accompanied by loss of function, is associated with nutrition imbalance, physical inactivity, insulin resistance, inflammation, metabolic syndrome, and atherosclerosis which are risk factors for cardiovascular disease. However, its association with outcomes after ischemic stroke has not been well-established. This study investigated whether functional outcomes of patients with acute ischemic stroke is associated with sarcopenia.

**Methods:**

Data were collected from 568 consecutive patients with acute ischemic stroke with National Institute of Health Stroke Scale 0–5 or transient ischemic attack who underwent bioelectrical impedance analysis between March 2018 and March 2021. Sarcopenia was defined, as low muscle mass, as measured by bioelectrical impedance analysis, and low muscle strength, as indicated by the Medical Research Council score. Unfavorable functional outcome was defined as mRS score of 2–6 at 90 days after discharge. The relationship between functional outcomes and the presence of sarcopenia or its components was determined.

**Results:**

Of the 568 patients included (mean age 65.5 ± 12.6 years, 64.6% male), sarcopenia was detected in 48 (8.5%). After adjusting for potential confounders, sarcopenia was independently and significantly associated with unfavorable functional outcome (odds ratio 2.37, 95% confidence interval 1.15–4.73 for unfavorable functional outcome, odds ratio 2.10, 95% confidence interval 1.18–3.71 for an increase in the mRS score). Each component of sarcopenia was also independently associated with unfavorable functional outcome (odds ratio 1.76, 95% confidence interval 1.05–2.95 with low muscle mass, odds ratio 2.64, 95% confidence interval 1.64–4.23 with low muscle strength). The impact of low muscle mass was larger in men than in women, and in patients with lower muscle mass of the lower extremities than in those with lower muscle mass of the upper extremities.

**Conclusions:**

In this study, the prevalence of sarcopenia in patients with stroke was lower than most of previous studies and patients with sarcopenia showed higher likelihood for unfavorable functional outcomes at 90 days after acute ischemic stroke or TIA. Further investigation of the interventions for treating sarcopenia and its impact on the outcome of ischemic stroke patients is needed.

## Introduction

Stroke is one of the leading causes of disability worldwide, especially in the elderly (1). The proportion of disability at 3 months after ischemic stroke varied from 32.4 to 49.2% high enough to be a great burden to society (2). Although stroke severity and age are known to be strong determinants of outcome in patients with stroke (2), further efforts have been made to identify other potential factors for outcomes after a stroke. A better understanding of stroke outcomes is necessary to further reduce the burden of stroke and improve outcomes.

Sarcopenia, a loss of muscle mass accompanied by loss of function, is common in the older population, ranging from a prevalence of 3–52% (3, 4). Sarcopenia is associated with nutrition imbalance, physical inactivity, insulin resistance, inflammation, metabolic syndrome, and atherosclerosis (4). Because these conditions have been reported to play significant roles in the development of vascular diseases, patients with sarcopenia may be at an increased risk of cardiovascular disease or peripheral arterial disease (PAD) (5, 6). In addition, considering the potential linkage between functional outcome and low muscle mass or low muscle strength in patients with stroke (7–10), the presence of sarcopenia could be associated with unfavorable functional outcomes in patients with ischemic stroke. However, there is very limited information on the relationship between sarcopenia and functional outcomes after ischemic stroke.

In this study, the presence of sarcopenia was hypothesized as an independent risk factor for unfavorable functional outcomes in patients with ischemic stroke. This study aimed to investigate whether the functional outcome of ischemic stroke was associated with sarcopenia or the degree of muscle mass and muscle strength. In addition, we also investigated the impact of each upper and lower extremities muscle mass deficit separately, because the functional outcome could be affected by the location of skeletal muscle mass deficit as well as amount of skeletal muscle mass.

## Methods

### Study Population

This was a retrospective, hospital-based observational study. Data were collected from the Yonsei Stroke Cohort (Clinicaltrials.gov, NCT03510312), a hospital-based observational cohort study investigating the long-term prognosis of patients admitted to the Department of Neurology at Severance Stroke Center located in Seoul, Republic of Korea for acute ischemic stroke or transient ischemic attack within 7 days of symptom onset (11). All patients underwent brain computed tomography and/or magnetic resonance imaging and were thoroughly evaluated to determine their demographic data, medical history, clinical manifestations, vascular risk factors, and comorbidities. The patients also underwent standard blood tests, angiographic studies, and cardiac evaluation, such as transthoracic/transesophageal echocardiography and continuous electrocardiogram or Holter monitoring, unless they could not be performed because of the patient's poor condition or refusal to receive such examinations. Since March 2018, bioelectrical impedance analysis (BIA) has been routinely performed for patient could stand without assistance. Therefore, the patients who had premorbid modified Rankin Scale (mRS) 0–1 and presented with initial National Institute of Health Stroke Scale (NIHSS) of 0–5 were included in this study.

For this study, out of 2,346 patients admitted between March 2018 and March 2021, we excluded 237 patients who had premorbid disability (mRS of 2–5), 752 who had initial NIHSS > 5, 755 who did not undergo BIA due to cognitive problem or unstable neurological or medical condition, 13 who were not followed up until 90 days, and 21 with insufficient laboratory data. Finally, 568 patients were included in the study (Supplementary Figure 1).

This study was approved by the institutional review board of Severance Hospital, Yonsei University Health System and the requirement for informed consent was waived.

### Diagnosis of Sarcopenia

Sarcopenia was defined as having both low muscle strength and low muscle mass according to Asian Working Group for Sarcopenia (AWGS) algorithm for sarcopenia (12). Appendicular skeletal muscle mass, defined as summation of the muscle mass of four limbs, and body fat mass were measured using InBody 770 (InBody, Seoul, Korea) during admission. The BIA was measured during admission. Mean duration from hospitalization to measurement was 3.2 ± 2.6 days and those from symptom onset was 4.2 ± 2.6 days. The appendicular skeletal muscle mass index [appendicular skeletal muscle mass (kg)/height^2^ (m^2^), ASMI] measured by BIA was dichotomized by the value of 7.0 kg/m^2^ in men and 5.7 kg/m^2^ in women to define the presence of low muscle mass, which was also based on the definition of the AWGS (12). To measure skeletal muscle strength, the AWGS recommends the use of handgrip strength. However, the Medical Research Council (MRC) score was instead used because skeletal muscle strength was reported to correlate with the MRC score as well as handgrip strength among stroke patients with disabilities, such as arm weakness or ataxia (13, 14). MRC is the tool for evaluating muscle strength of three groups of muscle in all four limbs. A score from 0 (total paralysis) to 5 (normal strength) is assigned to each of them and sum of score is ranging from 0 to 60. The summation of the MRC score of bilateral shoulder abductors, elbow flexors, wrist extensors, hip flexors, knee extensors, and ankle dorsiflexors was measured at admission and dichotomized by the value of 54 for men and 53.42 for women to define low muscle strength (13).

### Clinical and Laboratory Data

Baseline characteristics, vascular risk factors, and comorbidities, including hypertension, diabetes mellitus, dyslipidemia, coronary arterial occlusive disease (CAOD), PAD, atrial fibrillation, and history of previous stroke, were collected. Current smoking and alcohol consumption statuses were also assessed. Heavy alcohol consumption was defined as >4 drinks on any single day or >14 drinks per week for men and as >3 drinks on any single day or >7 drinks per week for women (15). The initial stroke severity was assessed by stroke neurologists using the NIHSS. Stroke subtypes were classified according to the Trial of Org 10172 in the Acute Stroke Treatment classification (16).

On admission, venous blood samples were collected within 24 h of hospitalization. Laboratory data, including total cholesterol, triglyceride, high-density lipoprotein, low-density lipoprotein, glycated hemoglobin, blood urea nitrogen, creatinine, albumin, white blood cell count, and hemoglobin, were collected. Body weight and height were measured at admission, and the body mass index (BMI) was calculated. BMI was dichotomized by a value of 23 kg/m^2^ to define the obese status (17).

### Functional Outcome

We obtained data on functional outcomes for all patients from the Yonsei Stroke Cohort study where stroke neurologists and research nurses regularly contacted the patients or their caregivers during follow-up via regular face-to-face visits or telephone interviews with or without a medical chart review. This was to investigate the functional outcome at 90 days and the occurrence of mortality, vascular events (clinical stroke or acute coronary events), cancer, or risk factors newly detected during long-term follow-up. Functional outcome was evaluated using the mRS, and unfavorable functional outcome was defined as an mRS score of 2–6 at 90 days after discharge (18).

### Statistical Analyses

Clinical and laboratory variables between groups were compared using the *t*-test or Mann-Whitney *U*-test for continuous variables and the Chi-square test or Fischer's exact test for categorical variables as appropriate. Continuous and categorical variables are described as mean ± standard deviation or median (quartile) and as number (percent), respectively. Univariable and multivariable logistic regression analyses and ordinal regression analyses were used to assess the significant factors for unfavorable functional outcome and an mRS score shift. Since the distribution of ASMI and cut-off value for diagnosing low muscle mass were different per sex (Supplementary Figure 2), *Z*-transformed ASMI values in each sex group were used in the regression analysis. In the analysis of multivariable analysis, well-known factors associated with functional outcome after stroke, such as age, stroke severity and albumin levels were used as covariates (2). The initial NIHSS score did not enter the multivariable model including muscle strength, because of collinearity between the NIHSS score and sum of muscle strength. Subgroup analysis was performed to investigate whether the association between sarcopenia and outcome would differ according to age, sex, risk factors for ischemic stroke, stroke mechanism, and BMI. Flexible regression models with restricted cubic splines were also used to assess the association between functional outcome and ASMI or the sum of MRC. ASMI values were further grouped as lower extremity ASMI and upper extremity ASMI to evaluate the effect of low muscle mass in each extremity on functional outcome after stroke. Akalike information criteria were used to determine the number of knots (19) which was 3 for ASMI and 4 for mRS. Variables adjusted in the multivariable analysis were also used in flexible regression models for precise prediction. Statistical analysis was conducted using R package version 4.0.5 (http://www.R-project.org). The *P*-values were two-sided, and statistical significance was set at *p* < 0.05.

## Results

The mean age of the 568 patients was 65.5 ± 12.6 years, and 367 (64.6%) were men. The mean BMI was 24.2 ± 3.2, and the median initial NIHSS score was 2 (interquartile range 1, 3) and 52 patients received intravenous thrombolysis. Other baseline characteristics are presented in Table 1.

**Table 1:** Comparison of baseline characteristics by the presence of sarcopenia.

	**Total patients (*n* = 568)**	**Sarcopenia (–) (*n* = 520)**	**Sarcopenia (+) (*n* = 48)**	***P*-value**
Age, years	65.5 ± 12.6	65.0 ± 12.7	70.6 ± 9.7	0.001
Sex, male	367 (64.6%)	326 (62.7%)	41 (85.4%)	0.003
Hypertension	402 (70.4%)	363 (69.8%)	39 (81.2%)	0.108
Diabetes mellitus	162 (28.5%)	143 (27.5%)	19 (39.6%)	0.108
Dyslipidemia	204 (35.9%)	190 (36.5%)	14 (29.2%)	0.389
CAOD	146 (25.7%)	132 (25.4%)	14 (29.2%)	0.688
PAD	16 (2.8%)	16 (3.1%)	0 (0.0%)	0.437
Atrial fibrillation	77 (13.6%)	71 (13.7%)	6 (12.5%)	0.998
Previous stroke	94 (16.5%)	86 (16.5%)	8 (16.7%)	1.000
Current smoker	123 (22.7%)	112 (21.5%)	11 (22.9%)	0.969
Heavy drinker	123 (21.7%)	114 (21.9%)	9 (18.8%)	0.743
Stroke mechanism				0.713
Small vessel occlusion	90 (15.8%)	82 (15.8%)	8 (16.7%)	
Large–artery atherosclerosis	81 (14.3%)	72 (13.8%)	9 (18.8%)	
Cardio embolism	123 (21.7%)	115 (22.1%)	8 (16.7%)	
Others	274 (48.2%)	251 (48.3%)	23 (47.9%)	
Initial NIHSS, median (IQR)	2 (1, 3)	2 (1, 3)	4 (2, 5)	<0.001
Intravenous thrombolysis	52 (9.2%)	47 (9.0%)	5 (10.4%)	0.956
Total cholesterol (mmol/L)	4.4 ± 1.1	4.4 ± 1.1	4.3 ± 1.3	0.564
Triglyceride (mmol/L)	1.4 ± 0.8	1.4 ± 0.8	1.2 ± 0.6	0.098
High density lipoprotein (mmol/L)	1.2 ± 0.3	1.2 ± 0.3	1.2 ± 0.4	0.606
Low density lipoprotein (mmol/L)	2.7 ± 1.1	2.7 ± 1.0	2.7 ± 1.2	0.683
Glycated hemoglobin (%)	6.1 ± 1.2	6.1 ± 1.1	6.4 ± 1.4	0.080
Arterial brachial index	1.1 ± 0.1	1.1 ± 0.1	1.1 ± 0.1	0.333
White blood cell count (10^9^/L)	7.7 ± 2.7	7.7 ± 2.6	8.2 ± 3.2	0.259
Hemoglobin (g/L)	8.6 ± 1.2	8.6 ± 1.2	8.3 ± 1.2	0.069
Blood urea nitrogen (mmol/L)	6.2 ± 2.6	6.2 ± 2.6	6.1 ± 2.7	0.722
Creatinine (μmol/L)	90.6 ± 87.0	91.1 ± 89.3	85.0 ± 57.1	0.509
Albumin (g/L)	43.7 ± 4.0	43.7 ± 4.0	43.8 ± 3.6	0.779
BMI (kg/m^2^)	24.2 ± 3.2	24.5 ± 3.1	21.2 ± 2.4	<0.001
Body fat mass (kg)	17.6 ± 6.2	17.9 ± 6.3	13.8 ± 4.3	<0.001
Low muscle mass	147 (25.9%)	99 (19.0%)	48 (100%)	<0.001
Low muscle strength	182 (32.0%)	134 (25.8%)	48 (100%)	<0.001
ASMI (kg/m^2^)	7.0 ± 1.1	7.1 ± 1.1	6.3 ± 0.7	<0.001
Z transformed ASMI		0.1 ± 0.9	−1.3 ± 0.7	<0.001
Sum of MRC	60 (54, 60)	60 (54, 60)	54 (51, 54)	<0.001

*Data are presented as mean (standard deviation), median (IQR), or number (percentage)*.

*CAOD, coronary arterial occlusive disease; PAD, peripheral arterial disease; NIHSS, National Institute of Health Stroke Scale; BMI, body mass index; ASMI, appendicular skeletal muscle index; MRC, Medical Research Council*.

Forty-eight (8.5%) patients satisfied the criteria for sarcopenia. Low skeletal muscle mass was detected in 147 patients (25.9%) and low muscle strength in 182 (32.1%). Sarcopenia was more frequent in men (11.2%, 41/367) than in women (3.5%, 7/201) (*p* = 0.003). In the comparison of baseline characteristics between patients with and without sarcopenia, patients with sarcopenia were more likely to be older or male with a higher NIHSS score, lower BMI, and lower body fat mass at presentation. However, no significant differences were found in other clinical variables between the two groups (Table 1).

### Association Between Sarcopenia and Functional Outcome

**Figure 1:**
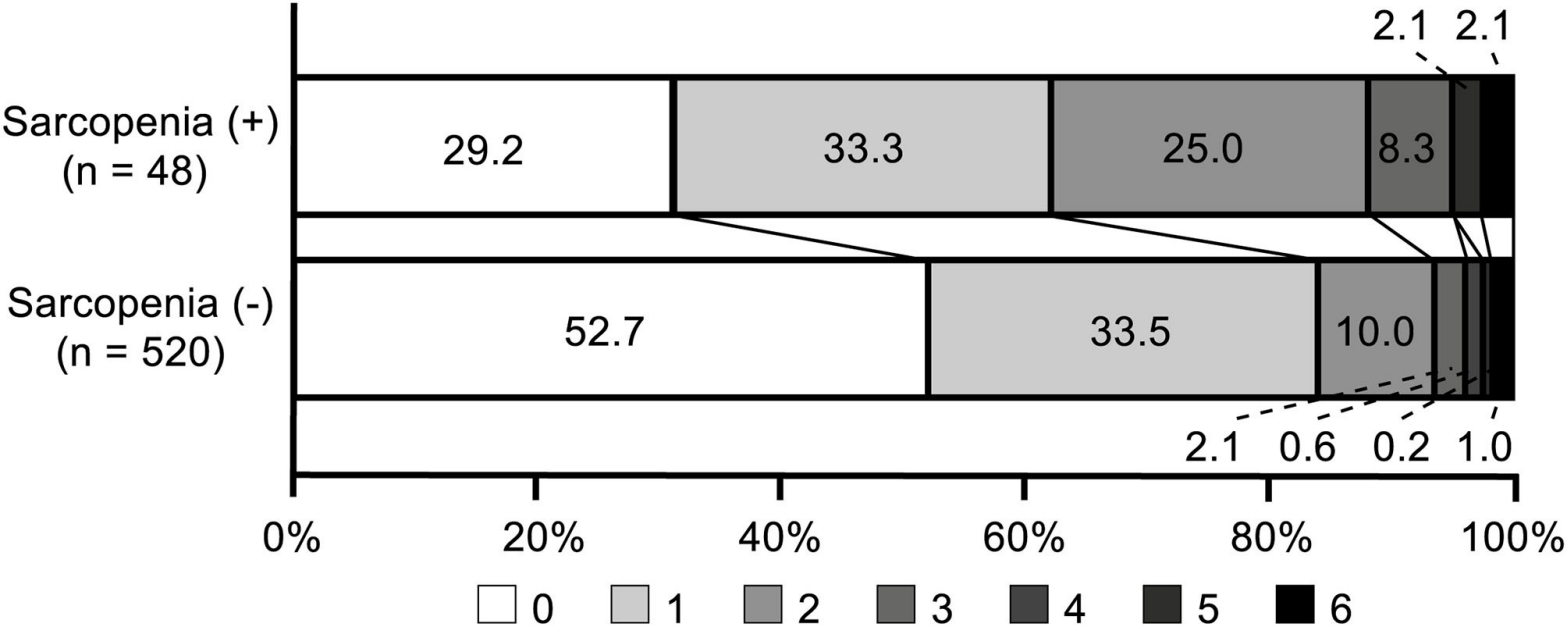
Distribution of modified Rankin Scale scores at 90 days in patients with and without sarcopenia.

**Figure 2:**
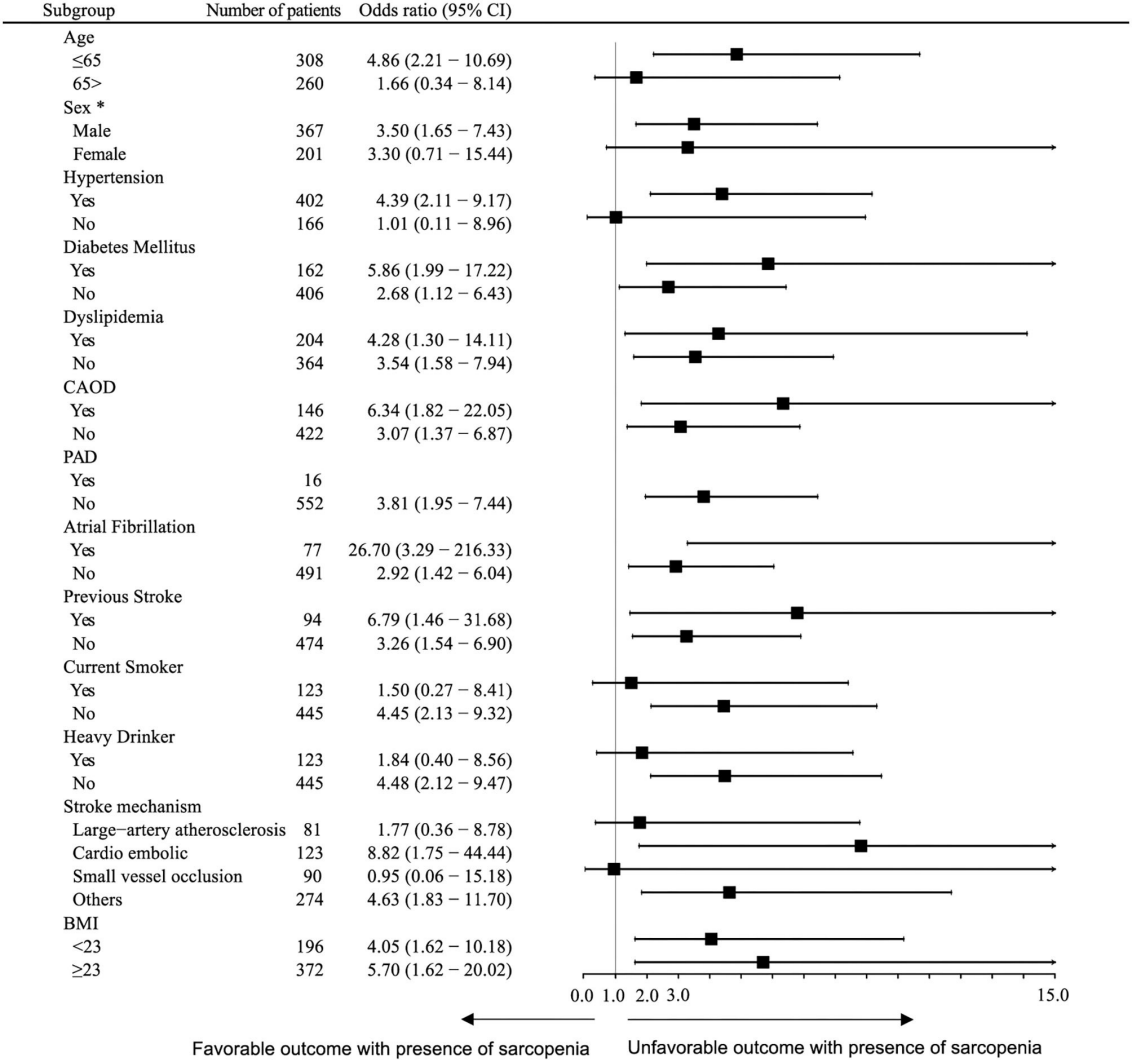
Subgroup analysis. Logistic regression analysis of the association between sarcopenia with unfavorable outcomes 90 days after discharge, adjusted for sex and age. *Adjusted for age only. CI, confidence interval; NIHSS, national institute of health stroke scale.

There were 90 (15.8%) patients who had unfavorable functional outcomes, and six (1.1%) patients died within 90 days after stroke. Figure 1 shows the distribution of the mRS score at 90 days after stroke in patients with and without sarcopenia. The proportion of patients with unfavorable functional outcome was larger in patients with sarcopenia than in those without (37.5 vs. 13.8%, *p* < 0.001), whereas the mortality was not significantly different between the two groups (2.1 vs. 1.0%, *p* = 1.000). Univariable logistic regression analysis showed that unfavorable functional outcome was associated with the presence of sarcopenia [odds ratio (OR) 3.73, 95% confidence interval (CI) 1.98–7.05, *p* < 0.001], along with older age, presence of hypertension, low level of hemoglobin or albumin, higher initial NIHSS score, and stroke mechanism of large artery atherosclerosis (all *p* < 0.05) (Table 2). After adjusting for the well-known factors associated with functional outcome after stroke, the presence of sarcopenia was an independent and significant predictor of unfavorable functional outcome (OR 2.37, 95% CI 1.15–4.73, *p* = 0.007) (Table 3). The effect of the presence of sarcopenia on unfavorable functional outcomes showed a consistent trend in diverse subgroups (Figure 2).

**Table 2:** Univariable logistic regression analysis and ordinal regression analysis for factors associated with functional outcome 90 days after discharge.

	**Unfavorable outcome—Logistic regression**		**mRS shift—Ordinal regression**	
	**Odds ratio (95% CI)**	***P*-value**	**Common odds ratio (95% CI)**	***P*-value**
Age, years	1.04 (1.02–1.07)	<0.001	1.03 (1.01–1.04)	<0.001
Sex (male)	0.67 (0.42–1.06)	0.087	0.92 (0.66–1.28)	0.618
Hypertension	1.94 (1.11–3.41)	0.020	1.49 (1.05–2.11)	0.026
Diabetes mellitus	1.23 (0.76–2.00)	0.397	1.22 (0.86–1.72)	0.266
Dyslipidemia	1.04 (0.65–1.66)	0.871	1.17 (0.85–1.62)	0.345
CAOD	1.56 (0.96–2.54)	0.073	0.98 (0.68–1.41)	0.901
PAD	1.23 (0.34–4.42)	0.747	1.32 (0.52–3.32)	0.561
Atrial fibrillation	1.34 (0.73–2.48)	0.349	0.80 (0.50–1.29)	0.360
Previous stroke	1.01 (0.55–1.85)	0.974	1.15 (0.76–1.74)	0.513
Current smoker	0.56 (0.30–1.06)	0.073	0.87 (0.60–1.27)	0.467
Heavy drinker	1.12 (0.66–1.92)	0.674	1.15 (0.79–1.68)	0.453
Stroke mechanism
Small vessel occlusion	1		1	
Large–artery atherosclerosis	3.63 (1.44–9.18)	0.006	1.36 (0.85–2.15)	0.191
Cardio embolism	2.44 (0.99–6.02)	0.052	0.68 (0.45–1.04)	0.077
Others	2.12 (0.96–5.10)	0.064	0.81 (0.52–1.27)	0.358
Initial NIHSS	1.41 (1.23–1.62)	<0.001	1.35 (1.23–1.49)	<0.001
Total cholesterol (mmol/L)	0.94 (0.77–1.16)	0.590	1.05 (0.91–1.21)	0.484
Triglyceride (mmol/L)	1.06 (0.80–1.41)	0.675	1.23 (1.01–1.49)	0.041
High density lipoprotein (mmol/L)	0.87 (0.41–1.83)	0.717	0.91 (0.54–1.53)	0.720
Low density lipoprotein (mmol/L)	0.94 (0.76–1.16)	0.556	1.03 (0.89–1.20)	0.656
Glycated hemoglobin (%)	1.06 (0.89–1.28)	0.509	1.09 (0.96–1.23)	0.191
Arterial brachial index	0.25 (0.05–1.29)	0.098	0.20 (0.06–0.71)	0.013
White blood cell count (10^9^/L)	0.98 (0.90–1.07)	0.605	0.98 (0.92–1.04)	0.512
Hemoglobin (g/L)	0.76 (0.63–0.91)	0.003	0.90 (0.78–1.02)	0.104
Blood urea nitrogen (mmol/L)	1.00 (0.92–1.09)	0.981	0.95 (0.89–1.01)	0.095
Creatinine (μmol/L)	1.00 (0.99–1.00)	0.414	1.00 (0.99–1.00)	0.025
Albumin (g/L)	0.90 (0.85–0.96)	0.001	0.95 (0.91–0.99)	0.008
BMI (kg/m^2^)	0.97 (0.90–1.04)	0.399	0.99 (0.94–1.04)	0.564
Body fat mass (kg)	1.01 (0.97–1.04)	0.753	1.00 (0.98–1.03)	0.745
Sarcopenia	3.73 (1.98–7.05)	<0.001	3.19 (1.83–5.56)	<0.001
Low muscle mass	2.62 (1.64–4.19)	<0.001	1.41 (0.98–2.03)	0.062
Low muscle strength	2.63 (1.66–4.16)	<0.001	2.29 (1.64–3.21)	<0.001
*Z*-transformed ASMI	0.69 (0.55–0.86)	0.001	0.83 (0.71–0.98)	0.025
Sum of MRC	0.80 (0.75–0.86)	<0.001	0.85 (0.81–0.89)	<0.001

*mRS, modified Rankin Scale; CI, confidence interval; CAOD, coronary arterial occlusive disease; PAD, peripheral arterial disease; NIHSS, National Institute of Health Stroke Scale; BMI, body mass index; ASMI, appendicular skeletal muscle index; MRC, Medical Research Council*.

**Table 3:** Multivariable analysis for the association between sarcopenia and functional outcomes 90 days after discharge.

	Logistic regression analysis^*^		Ordinal regression analysis^*^	
	**Odds ratio (95% CI)**	***P*-value**	**Odds ratio (95% CI)**	***P*-value**
Sarcopenia	2.37 (1.15–4.73)	0.007	2.10 (1.18–3.71)	0.011
Low muscle mass	1.76 (1.05–2.95)	0.032	1.03 (0.69–1.52)	0.898
Low muscle strength	2.64 (1.64–4.23)	<0.001	2.26 (1.61–3.17)	<0.001

* *Adjusted for age, albumin, and initial NIHSS score. CI, confidence interval*.

Further, ordinal regression analysis was performed to determine a significant variable for a shift in the mRS score at 90 days after discharge. Univariable and multivariable ordinal regression analysis showed that the presence of sarcopenia (OR 2.10, 95% CI 1.18–3.71, *p* = 0.011) was a significant and independent predictor for an increase in the mRS score at 90 days after discharge (Table 3).

### Association Between Each Component of Sarcopenia and Functional Outcome

We investigated which component of sarcopenia was independently associated with unfavorable functional outcomes. Univariable and multivariable logistic regression analyses showed that all components of sarcopenia, either the presence of low muscle mass (OR 1.76, 95% CI 1.05–2.95, *p* = 0.032) or low muscle strength (OR 2.64, 95% CI 1.64–4.23, *p* < 0.001), were independently associated with unfavorable functional outcome (Tables 2, 3). Functional outcome was also different according to the severity of each component of sarcopenia in that the mRS score at 90 days after discharge increased with the elevation of the MRC score or *Z*–transformed ASMI (Supplementary Figure 3).

Likewise, in univariable and multivariable ordinal regression analyses, low muscle strength (OR 2.26, 95% CI 1.61–3.17, *p* < 0.001) was independently associated with an mRS score shift. The presence of low muscle mass (OR 1.03, 95% CI 0.69–1.52, *p* = 0.898) showed a trend toward an mRS score shift, although the difference was not statistically significant (Table 3).

### Differential Impact of Sarcopenia on Functional Outcome by Sex or Location of Lower Muscle Mass

**Figure 3:**
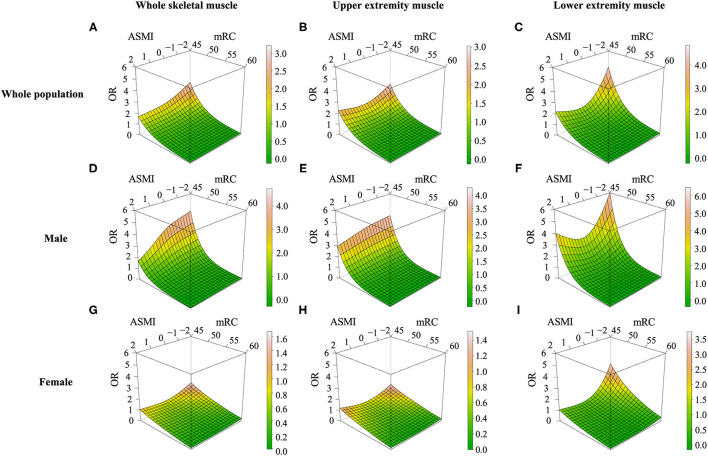
Restricted cubic spline models for low muscle mass, low muscle strength, and unfavorable outcomes at 90 days. Effect of low muscle mass and low muscle strength on functional outcomes in ischemic stroke or TIA patients assessed with **(A,D,G)** *Z*-transformed appendicular skeletal muscle mass index, **(B,E,H)** only upper extremity muscle mass **(C,F,I)** only lower extremity muscle mass. OR, odds ratio; ASMI, *Z*-transformed appendicular skeletal muscle index; MRC, Medical Research Council.

Restricted cubic spline models adjusting for age, albumin, and initial NIHSS demonstrated that the probability of unfavorable functional outcome was highest in case of having both lower *Z*-transformed ASMI value and low sum of MRC. In addition, the impact of lower muscle mass, lower muscle strength, or both appeared smaller in female than in male (Figure 3). When we investigated whether functional outcome was differently affected by the location of low skeletal muscle mass assessed with *Z*-transformed value of each lower and upper extremity ASMI, functional outcomes were more dependent on muscle mass of the lower extremities rather than that of the upper extremities. Low muscle mass in both upper and lower extremities showed greater influence on unfavorable functional outcome in the male group, similar to those of the whole appendicular muscle (Figure 3).

## Discussion

This study showed that the presence of sarcopenia, defined using low muscle mass and low muscle strength, was significantly associated with unfavorable functional outcomes in patients with mild acute ischemic stroke and TIA. This relationship was consistently observed in the diverse subgroups. Moreover, each component defining sarcopenia was independently associated with unfavorable outcomes. The impact of low muscle mass was larger in male than in female and in patients with lower muscle mass of the lower extremities than in those with lower muscle mass of the upper extremities.

In this study, sarcopenia was detected in 8.5% of patients with mild acute ischemic stroke and TIA. Although the prevalence of sarcopenia among the stroke population varies (8, 9, 20, 21), several studies showed that sarcopenia among stroke patients were detected in 53–62%, which were higher than that of our study (9, 21). This difference could be caused by various factors. The prevalence of sarcopenia, an age–related disease, is expected to be high in the elderly population (4). This trend was also evident in the current study population, with the frequency of sarcopenia increased from 2.9% in patients aged < 50 years to 12.0% in those aged > 70 years (Supplementary Figure 4). In addition, several studies conducting stroke population used only low handgrip strength (7, 8) or low skeletal muscle mass (9) to define the sarcopenia, which may have led to the overestimation. In this study, the prevalence of low muscle mass and low muscle strength were 25.9 and 32.0%, respectively. Moreover, only patients with mild ischemic stroke and TIA without premorbid disability were enrolled in this study. These differences among studies could be related to the wide variability in the prevalence of sarcopenia in the stroke population.

Although the presence of sarcopenia is a well-known risk factor for unfavorable functional outcome in patients with vascular disease or metabolic syndrome (4, 22), the impact of sarcopenia on functional outcomes after stroke is not well-known. Previous studies including a small number of patients with stroke patients demonstrated the negative effect of handgrip strength or skeletal muscle mass on the functional outcome at discharge or within 3 weeks after stroke (7–9). Others showed that not just sarcopenia, but sarcopenia with slow walking speed or only severe degree of muscle mass deficit showed its association with disability (23, 24). In this study, we focused the functional outcome after 90 days using a relatively large number of acute stroke patients with each component of sarcopenia, as well as the presence of sarcopenia itself. As results, our analysis also showed that each component of sarcopenia, as well as the presence of sarcopenia itself had a separate negative impact on the functional outcome in stroke survivors.

There are several possible explanations for the association between sarcopenia and unfavorable functional outcomes. First, the difference in outcomes may be related to baseline characteristics, initial stroke severity, or comorbidities between sarcopenic and non-sarcopenic patients. However, sarcopenia remained a significant factor for unfavorable outcomes even after adjusting for potential confounders, and the detrimental effect of sarcopenia on the functional outcomes was still observed regardless of patient characteristics. Instead, malnutrition may be one of the factors that can explain the unfavorable outcomes of patients with sarcopenia in mild acute stroke or TIA. A previous study showed that sarcopenia also affect tongue muscles; thus, oral sarcopenia may be related to poor oral status (25). This poor oral status of sarcopenia patients could subsequently lead to a malnutritional status and unfavorable outcome in patients with ischemic stroke.

We also demonstrated the association between sarcopenia and stroke outcome were more evident in men than in women. Previous reports have suggested that the linkage between sarcopenia and the mortality and physical limitation was more evident in men than in women (26). Further, men were more likely to have sarcopenic obesity (a combination of sarcopenia and obesity), which had a worse impact on the outcome of vascular or metabolic disease compared with sarcopenia without obesity (27, 28). These sex-dependent susceptibilities may be caused by a marked decline in insulin-like growth factor-1, one of the key factors in the pathophysiology of sarcopenia (29), which was more prominent in men than in women.

In addition, sarcopenia had a different clinical significance according to the location of skeletal muscle mass deficit. In this study, decreased muscle mass in the lower extremities had a greater impact on outcomes than that in the upper extremities. Considering physical disability could be dependent on the degree of total skeletal muscle mass deficit (24), and lower extremity muscle mass is relatively larger than that of the upper extremity, it seems reasonable that lower extremity muscle mass deficit showed a greater impact on recovery after stroke. In addition, lower extremity muscle mass may be associated with sedentary behavior. Physical inactivity related to sedentary behavior did not allow the retention of lower extremity strength or muscle mass, and subsequently, led to an incomplete recovery after stroke. In addition, concomitant clinical or subclinical PAD could involve lower extremity muscle mass deficits. PAD has been recognized as a predictor of poor outcomes in patients with ischemic stroke and poor physical activity may elevate the risk of hidden PAD (30). Along with these issues, our data suggested location-specific differences in the effect of lower skeletal muscle mass deficit.

This study has several limitations. First, only patients who could stand unassisted and had relatively mild stroke severity or patients with TIA were included; therefore, the results cannot be generalized to all patients with stroke. Second, the data of patients were retrospectively derived from a single stroke center, although patients were consecutively registered in the stroke registry. All patients with stroke were managed based on current updated guidelines and protocolized care at the researchers' stroke center. Third, the sum of MRC was used as a representative of muscle strength, which is not included in the recommended methods of low muscle mass in the AWGS protocol. However, as mentioned before, the AWGS protocol did not consider specific conditions, such as acute ischemic stroke, and the use of MRC instead of using handgrip strength has been validated (13, 14). Finally, the presence of low muscle mass defined according to AWGS guideline. Thus, the result could not be generalized to all patients, especially non-Asians.

## Conclusion

This study showed that the presence of sarcopenia was independently associated with unfavorable functional outcomes in patients with mild acute ischemic stroke and TIA. These relationships were more evident in men and in cases of low muscle mass in the lower extremities. These findings shed new light on the clinical significance of sarcopenia in patients with stroke. Further investigation of interventions for treating sarcopenia and its impact on the outcome of ischemic stroke patients through serial assessment of sarcopenia is needed for better understanding.

## Data Availability Statement

The original contributions presented in the study are included in the article/Supplementary Material, further inquiries can be directed to the corresponding author/s.

## Ethics Statement

The studies involving human participants were reviewed and approved by the Institutional Review Board of Severance Hospital, Yonsei University Health System. Written informed consent for participation was not required for this study in accordance with the national legislation and the institutional requirements.

## Author Contributions

HL: acquisition of data, analysis and interpretation of data, and writing of original draft. IL, JH, MB, and HP: acquisition of data and interpretation of data. HSL: analysis and interpretation of data and critical revision of the manuscript for intellectual content. HN: interpretation of data and critical revision of the manuscript for intellectual content. YK: study concept and design, analysis and interpretation of data, and critical revision of the manuscript for intellectual content. All authors contributed to the article and approved the submitted version.

## Funding

This study was supported by a faculty research grant of Yonsei University College of Medicine (6-2020-0202, 6-2019-0191) and a grant of the Korea Health Technology R&D Project through the Korea Health Industry Development Institute (KHIDI), funded by the Ministry of Health & Welfare, Republic of Korea (HC19C0028).

## Conflict of Interest

The authors declare that the research was conducted in the absence of any commercial or financial relationships that could be construed as a potential conflict of interest.

## Publisher's Note

All claims expressed in this article are solely those of the authors and do not necessarily represent those of their affiliated organizations, or those of the publisher, the editors and the reviewers. Any product that may be evaluated in this article, or claim that may be made by its manufacturer, is not guaranteed or endorsed by the publisher.
