# Supplementary material for: Impact of Sarcopenia on Functional Outcomes Among Patients With Mild Acute Ischemic Stroke and Transient Ischemic Attack: A Retrospective Study
Source: Front Neurol. 2022 Mar 15;13:841945. doi: 10.3389/fneur.2022.841945 (PMC8964497; doi:10.3389/fneur.2022.841945)
Supplement: Supplementary file 1 [file Data_Sheet_1.docx]

**Supplementary materials (online only)**

**Impact of sarcopenia on functional outcomes among patients with mild acute ischemic stroke and transient ischemic attack : a retrospective study**

**Supplementary Table 1**. Multivariable logistic regression analysis for factors associated with functional outcome 90 days after discharge

|  | Model - 1 | | Model2 | | Model3 | | Model4 | | Model5 | |
| --- | --- | --- | --- | --- | --- | --- | --- | --- | --- | --- |
|  | Odds ratio (95% CI) | P-value | Odds ratio (95% CI) | P-value | Odds ratio (95% CI) | P-value | Odds ratio (95% CI) | P-value | Odds ratio (95% CI) | P-value |
| Age | 1.03 (1.01 – 1.05) | 0.007 | 1.03 (1.00 – 1.05) | 0.029 | 1.03 (1.01 – 1.06) | 0.002 | 1.03 (1.00 – 1.05) | 0.018 | 1.03 (1.01 – 1.05) | 0.010 |
| Albumin (g/L) | 0.92 (0.86 – 0.98) | 0.017 | 0.92 (0.87 – 0.98) | 0.014 | 0.92 (0.86 – 0.98) | 0.006 | 0.92 (0.87 – 0.99) | 0.017 | 0.91 (0.86 – 0.98) | 0.006 |
| Initial NIHSS | 1.34 (1.16 – 1.54) | <0.001 | 1.36 (1.19 – 1.57) | <0.001 |  |  | 1.38 (1.19 – 1.58) | <0.001 |  |  |
| Sarcopenia | 2.37 (1.15 – 4.73) | 0.014 |  |  |  |  |  |  |  |  |
| Low muscle mass |  |  | 1.76 (1.05 – 2.95) | 0.032 |  |  |  |  |  |  |
| Low muscle strength |  |  |  |  | 2.64 (1.64 – 4.23) | <0.001 |  |  |  |  |

All models were adjusted with age and albumin. Model 1, 2, and 4 were futher adjusted with initial NIHSS. CI, confidence interval; NIHSS, national institute of health stroke scale

**Supplementary Table 2.** Multivariable ordinal regression analysis for factors associated with functional outcome 90 days after discharge

|  | Model - 1 | | Model2 | | Model3 | | Model4 | | Model5 | |
| --- | --- | --- | --- | --- | --- | --- | --- | --- | --- | --- |
|  | Common Odds ratio (95% CI) | P-value | Common Odds ratio (95% CI) | P-value | Common Odds ratio (95% CI) | P-value | Common Odds ratio (95% CI) | P-value | Common Odds ratio (95% CI) | P-value |
| Age | 1.02 (1.00 – 1.03) | 0.009 | 1.02 (1.01 – 1.04) | 0.006 | 1.02 (1.01 – 1.03) | 0.007 | 1.02 (1.01 – 1.04) | 0.007 | 1.02 (1.00 – 1.03) | 0.022 |
| Albumin (g/L) | 0.97 (0.92 – 1.01) | 0.115 | 0.97 (0.93 – 1.01) | 0.143 | 0.96 (0.92 – 1.00) | 0.065 | 0.97 (0.93 – 1.01) | 0.145 | 0.96 (0.91 – 1.00) | 0.050 |
| Initial NIHSS | 1.32 (1.19 – 1.45) | <0.001 | 1.35 (1.22 – 1.49) | <0.001 |  |  | 1.35 (1.22 – 1.49) | <0.001 |  |  |
| Sarcopenia | 2.10 (1.18 – 3.71) | 0.011 |  |  |  |  |  |  |  |  |
| Low muscle mass |  |  | 1.03 (0.69 – 1.52) | 0.898 |  |  |  |  |  |  |
| Low muscle strength |  |  |  |  | 2.26 (1.61 – 3.17) | <0.001 |  |  |  |  |

All models were adjusted with age and albumin. Model 1, 2, and 4 were futher adjusted with initial NIHSS. CI, confidence interval; NIHSS, national institute of health stroke scale

**Supplementary Figure 1.** Flowchart of Study Population Selection

TIA, transient ischemic attack; NIHSS; national institute of health stroke scale; BIA, bioelectrical impedance analysis

**Supplementary Figure 2.** Distribution of ASMI (kg/m^2^) according to sex

Dashed line indicates AWGS 2019 guideline cut off value for diagnosis of low muscle mass in each sex. ASMI, appendicular skeletal muscle mass index

**Supplementary Figure 3.** Distribution of mRS 90 days after discharge by sum of MRC and ASMI

Distribution of mRS 90 days after discharge according to (A) sum of MRC (B) appendicular skeletal muscle mass index. MRC, Medical Research Council; ASMI, appendicular skeletal muscle index; mRS modified Rankin Scale

**Supplementary Figure 4.** Prevalence of sarcopenia and ASMI distribution according to age

Distribution of (A) prevalence of sarcopenia and (B) appendicular skeletal muscle mass index according to different age group. ASMI, appendicular skeletal muscle mass index.
